# Transcriptome Analysis Reveals the Accumulation Mechanism of Anthocyanins in Buckwheat (*Fagopyrum esculentum* Moench) Cotyledons and Flowers

**DOI:** 10.3390/ijms20061493

**Published:** 2019-03-25

**Authors:** Zhengwu Fang, Zehao Hou, Shuping Wang, Zhixiong Liu, Shudong Wei, Yingxin Zhang, Jinghan Song, Junliang Yin

**Affiliations:** 1 Hubei Collaborative Innovation Center for Grain Industry/Hubei Key Laboratory of Waterlogging Disaster and Agricultural Use of Wetland/College of Agriculture, Yangtze University, Jingzhou 434000, China; fangzhengwu88@163.com (Z.F.); 201771374@yangtzeu.edu.cn (Z.H.); wangshuping2003@126.com (S.W.); zhangyingxin1985@126.com (Y.Z.); songjingh@126.com (J.S.); 2 College of Horticulture and Gardening, Yangtze University, Jingzhou 434000, China; zxliu77@yahoo.com; 3 College of Life Science, Yangtze University, Jingzhou 434000, China; weishudong2005@126.com; 4 Forewarning and Management of Agricultural and Forestry Pests, Hubei Engineering Technology Center/Engendering Research Center of Ecology and Agricultural Use of Waterland, Ministry of Education, Yangtze University, Jingzhou 434000, China

**Keywords:** buckwheat (*Fagopyrum esculentum* Moench), cotyledon, flower, transcriptome analysis, anthocyanin

## Abstract

Buckwheat (*Fagopyrum esculentum*) is a valuable crop which can produce multiple human beneficial secondary metabolites, for example, the anthocyanins in sprouts and flowers. However, as the predominant group of visible polyphenols in pigmentation, little is known about the molecular mechanisms underlying the anthocyanin biosynthesis within buckwheat. In this study, a comparative transcriptome analysis of green and red common buckwheat cultivars was carried out through RNA sequencing. Overall, 3727 and 5323 differently expressed genes (DEGs) were identified in flowers and cotyledons, respectively. Through GO and KEGG analysis, we revealed that DEGs in flowers and cotyledons are predominately involved in biosynthesis of anthocyanin. A total of 42 unigenes encoding 11 structural enzymes of the anthocyanin biosynthesis were identified as DEGs. We also identified some transcription factor families involved in the regulation of anthocyanin biosynthesis. Real-time qPCR validation of candidate genes was performed in flowers and cotyledons, and the results suggested that the high expression level of structural genes involved in anthocyanin biosynthetic pathway promotes anthocyanin accumulation. Our results provide the insight understanding for coloration of red common buckwheat.

## 1. Introduction

As one of the most important grain and honey crops, buckwheat (*Fagopyrum esculentum* Moench) is widely planted all over the world, especially in China, Japan, Canada, and Russia [1,2]. The harvested seeds of buckwheat are conventionally used to make whole grain foods, groats, noodles, and flour, as well as other cereal crops [3,4]. It has been reported that buckwheat can accumulate abundant nutrients in grain and hull, such as flavonoids, anthocyanin, essential amino acids, and tannin [5,6,7]. Generally, common buckwheat has two different flower colors, either white or pink. However, some cultivars have developed red flowers, which is believed to be related to the accumulation of anthocyanin, and the petals of the red flower buckwheat are traditionally used as materials in the manufacturing of several beverages, such as alcohol and tea after the detoxification of fagopyrin [8,9].

Anthocyanin is normally considered to be one of the water-soluble flavonoids which are widely distributed in plant vegetative tissues, and are synthesized in cytoplasm then accumulated in vacuoles. The ranging colors, from red to purple, reflected on higher plant tissues are mostly attributed to the level of anthocyanin accumulation [10,11]. Moreover, anthocyanins play important roles in plant development, such as providing protection against UV radiation, low temperature, drought stress, and pathogens [12,13,14], and their accumulation in flowers attracts pollinators [15,16]. More importantly, these pigments could also help humans to prevent several chronic diseases [17,18].

The anthocyanin biosynthetic pathway has been extensively studied in several plant species, including *Petunia hybrid*, *Antirrhinum majus*, and *Zea mays* [19,20,21], and the biosynthesis of anthocyanin is viewed as a branch of flavonoid production The structural genes encoding multiple enzymes of this pathway are classified as two groups: the early-stage biosynthetic genes (EBGs), including *phenylalanine ammonia-lyase* (*PAL*), *chalcone synthase* (*CHS*), *chalcone isomerase* (*CHI*), *flavanone 3-hydroxylase* (*F3H*); and the late-stage biosynthetic genes (LBGs) induced afterwards, *dihydroflavonol reductase* (*DFR*), *anthocyanidin synthase*/*leucoanthocyanidin oxygenase* (*ANS/LDOX*), and *UDP-glucose: flavonoid 3-O-glucosyltransferase* (*UFGT*) are involved in the LBGs [22,23]. Transcriptional factors regulating the expression of these genes are presently known as R2R3 MYB, basic helix-loop-helix (bHLH), and WD repeat (WDR), which can form a MYB-bHLH-WDR (MBW) complex binding to the anthocyanin structural gene promoter to control its transcription and affect the accumulation of anthocyanins [24,25,26]. Currently, with the development of the 3rd-generation sequencing technology, an increasing number of genes involved in the plant anthocyanin biosynthetic have been identified and annotated without reference genomes, such as *Litchi chinensis* [27], bougainvillea [28], and radish [29]. Nevertheless, the molecular mechanism of pigment accumulation in red buckwheat has yet to be further explored.

A previous study has reported that a Chinese buckwheat variety, Gan-Chao, which accumulates anthocyanins in petals, that the accumulation increased along with the development of the flower [8,9]. Likewise, in our previous study, we discovered a local buckwheat cultivar, “HHTQ”, in the Guizhou province of China. The cultivar presents red cotyledons, leaves, and flowers during the growth, acting as a potentially favorable material to not only study the molecular biosynthesis of anthocyanin in buckwheat, but also provide the very opportunity to develop anthocyanin-rich buckwheat products. In this study, we have developed the high-throughput RNA sequencing (RNA-seq) in both cotyledon and flowers of the green and red buckwheat cultivars respectively, through which the structural genes involved in anthocyanin biosynthesis were systemically analyzed. Our study may improve understanding of the regulatory mechanism of buckwheat anthocyanin production.

## 2. Results

### 2.1. Anthocyanins Quantification

**Figure 1:**
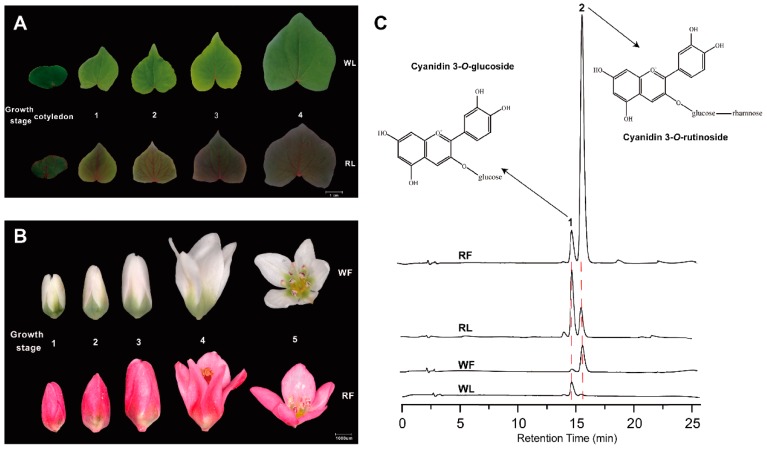
The morphology of buckwheat leaves and flowers, and component analysis of anthocyanins in different buckwheat cultivars. (**A**) The leaves of white cultivar “Beizaosheng” (WL) and red cultivar “HHTQ” (RL) at different growth stages. Scale bar = 1 cm. (**B**) The flowers of white cultivar “Beizaosheng” (WF) and red cultivar “HHTQ” (RF) at different growth stages. Scale bar = 1000 µm. (**C**) High-performance liquid chromatography (HPLC) profiles of anthocyanins extracted from the cotyledon and flowers of two buckwheat cultivars (detected at A520).

The leaves and flowers from two cultivars at different growth stages are shown in Figure 1. Apparently, cultivar “HHTQ” has red-vein cotyledons, red leaves, and red petals during the different growth stages. Comparatively, cultivar “Beizaosheng” presents green leaves and white flowers (Figure 1A,B. Origin figures can be found in Figure S1). To examine the anthocyanin composition in the buckwheat, the high-performance liquid chromatography (HPLC) was carried out and identified two major kinds of anthocyanins—cyanidin 3-*O*-gulcoside (peak 1) and cyanidin 3-*O*-rutinoside (peak 2)—in the cotyledon and flower. The highest levels of cyanidin 3-*O*-gulcoside and cyanidin 3-*O*-rutinoside were found in the cotyledon and flower of “HHTQ” (Figure 1C, Table 1). The total anthocyanin contents at flower and cotyledon of the “HHTQ” were 7.51-fold and 5.12-fold higher than that of “Beizaosheng”, respectively, and the red flower of “HHTQ” contained a higher amount of cyanidin 3-*O*-rutinoside (2.83 mg/g DW) than “Beizaosheng” (0.38 mg/g DW). In the cotyledon of “HHTQ”, cyanidin 3-*O*-gulcoside showed the highest content (0.97 mg/g DW), obviously higher than cyanidin 3-*O*-rutinoside, and there were not obvious absorption signals for cyanidin 3-*O*-rutinoside detected in the cotyledon of “Beizaosheng”. These results suggest that more anthocyanins are accumulated in red cultivar “HHTQ”, and the accumulation of cyanidin 3-*O*-rutinoside in flowers and the accumulation of cyanidin 3-O-gulcoside in cotyledon are higher.

### 2.2. Full-Length Transcriptome Sequencing and RNA-seq

**Figure 2:**
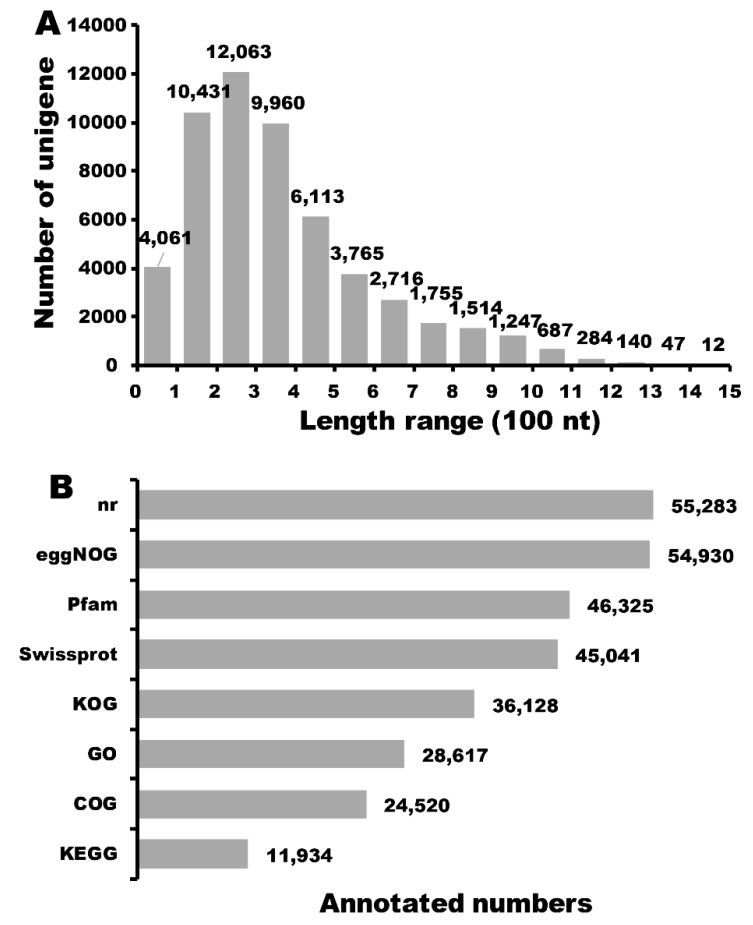
Predicted length distribution map of coding sequence (CDS) encoded protein and statistic summary of functional annotation for buckwheat unigenes in public databases. (**A**) Predicted length distribution map of CDS encoded protein. Nucleotide: (nt). (**B**) Statistic summary of functional annotation for buckwheat unigenes in eight public databases.

There is a reported genome of buckwheat (*Fagopyrum esculentum* Moench) [30] which is distantly related to the cultivars used in our study (*Fagopyrum tataricum* (L.) Gaertn). To fully understand the potential molecular mechanism involved in anthocyanin synthesis, we firstly performed the full-length transcriptome sequencing to three libraries with different size fractions (1–2 K, 2–3 K, and 3–6 K) on five SMRT cells using the PacBio Sequel system (Table S1). A total of 281,566 reads of insert (ROIs), including 126,054 ROIs from two SMRT cells for 1–2 K fractions, 91,798 ROIs from two SMRT cells for 2–3 K fractions, and 63,714 ROIs from a SMRT cell for 3–6 K fractions were generated with an average of 3,536 bp in length, and 123,039 full length reads non-chimeric (FLNC) remained after circular consensus sequence (CCS) generation and filtering for full-length read classification. Secondly, we performed Illumina RNA-Seq to twelve cDNA libraries and there were 22,322,429 (RF), 23,503,180 (WF), 23,413,984 (RL), and 23,218,940 (WL) clean reads left after trimming and filtering (Table S2). The full-length (FL) reads were clustered into consensus clusters based on RS_IsoSeq of SMRT Analysis, then 64,539 high-quality (HQ) and consensus isoforms were merged into 51,673 final consensus sequences in total, and 12,866 low-quality consensus isoforms were rectified using the Illumina RNA-seq data. Subsequently, we utilized CD-HIT for further clustering to obtain the non-redundant transcripts and a total of 56,864 full-length transcripts of *Fagopyrum esculentum* were generated. There were 37,881 complete ORFs that were obtained by TransDecoder software, and the length of CDS (coding sequence) encoded protein is shown in Figure 2A. Of these CDSs, only a minority (59 CDSs) were more than 1300 nucleotide (nt), and 70.39% of CDSs appeared with a length ranging from 100 to 500 nt. All unigenes were blasted against eight public databases: Nr, Swiss-Prot, KEGG, GO, KOG, Pfam, eggnog, and COG, and the results showed that 55,426 unigenes were annotated in this transcriptome data (Figure 2B). Among the 55,426 unigenes, 24,520 unigenes (44.24%), 36,128 unigenes (65.18%), 46,325 unigenes (83.58%), 55,283 unigenes (99.74%), 54,930 unigenes (99.11%), 45,041 unigenes (81.26%), 28,617 unigenes (51.63%), and 11,934 unigenes (21.53%) were annotated in COG, KOG, Pfam, Nr, eggNOG, Swissprot, GO, and KEGG, respectively.

### 2.3. Identification of Different Expressed Genes

**Figure 3:**
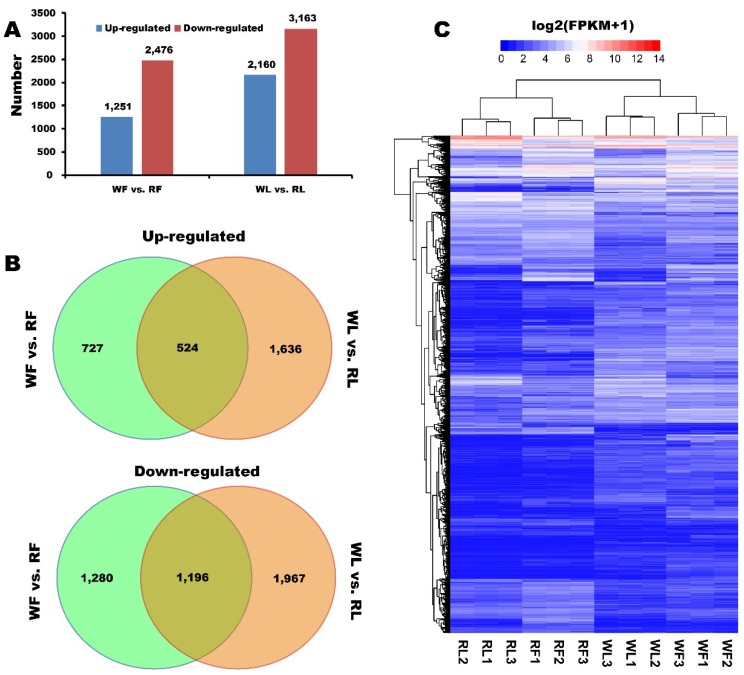
Changes in gene expression profiles in flowers and leaves of two different cultivars. (**A**) Numbers of differently expressed genes (DEGs) in pairwise comparisons in flowers and cotyledons of two different cultivars of buckwheat. (**B**) Venn diagram showing the number of DEGs in flowers and cotyledons of two different cultivars of buckwheat. (**C**) Heat-map diagrams showing the relative expression levels of total DEGs among the four tissues. WF vs. RF means the flowers sample of white cultivar “Beizaosheng” compared to red cultivar “HHTQ”, and RL vs. RL means the cotyledons samples of white cultivar “Beizaosheng” compared to red cultivar “HHTQ”. Up- and down-regulated means that genes are up- and down-regulated in “Beizaosheng” compared to “HHTQ”.

To further reveal the underlying molecular mechanisms of anthocyanin biosynthesis, we performed RNA-seq analysis to the cotyledon and flower materials of white cultivar “Beizaosheng” (WL and WF) and red cultivar “HHTQ” (RL and RF), and each has three replicates (Figure S2). A total of 9050 differently expressed genes (DEGs) in buckwheat were identified though pairwise comparisons, of which 3727 and 5323 DEGs were found between WF and RF, and WL and RL, respectively (Figure 3A). Comparison of the RF with the WF revealed 1251 unigenes that were up-regulated and 2476 unigenes that were down-regulated. In total, 2160 unigenes were up-regulated and 3163 genes were down-regulated under the RL compared with the WL. Five hundred and twenty-four unigenes of 1251 up-regulated DEGs between RF and WF were also among the genes differentially expressed between RL and WL. In addition, 1196 of 2476 down-regulated DEGs between RF and WF were also among the genes differentially expressed between RL and WL (Figure 3B). Furthermore, a general overview of the expression pattern was visualized in a heat map (Figure 3C), which provided an overall understanding of the change in gene expression.

### 2.4. GO Annotation, KEGG Pathway, and Enrichment Analyses

**Figure 4:**
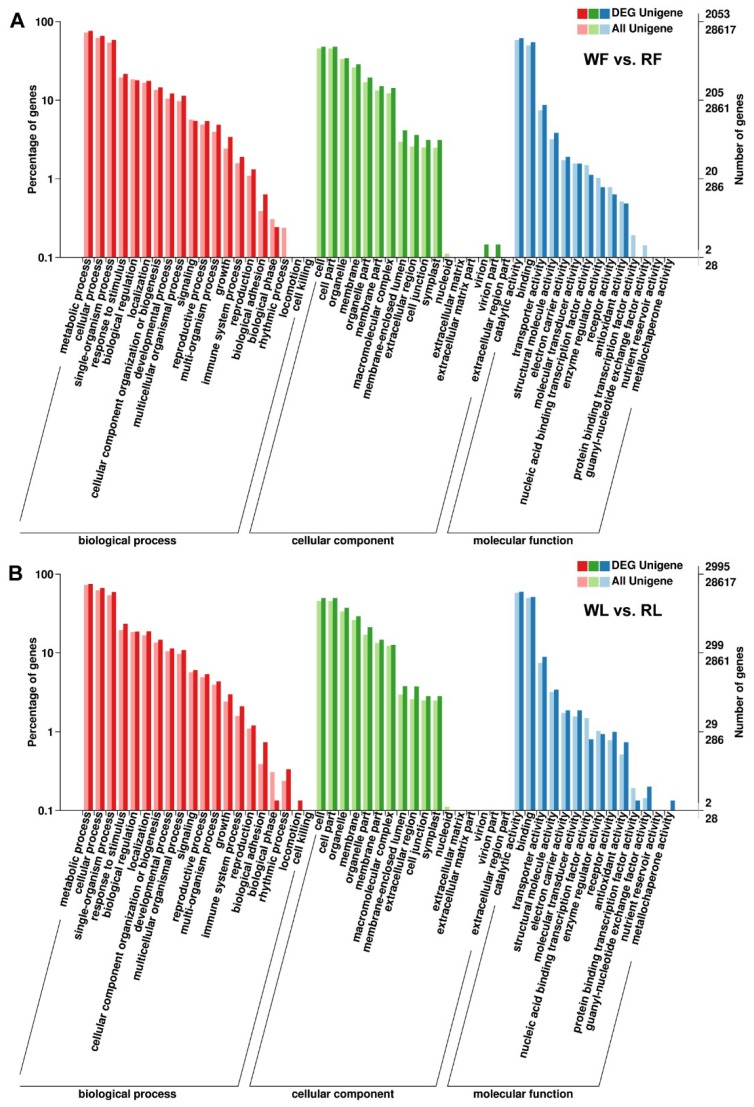
Gene ontology (GO) categorization of buckwheat unigenes. (**A**) GO analysis of DEGs between white flowers (WF) and red flowers (RF) in three main categories. (**B**) GO analysis of DEGs between white cotyledon (WL) and red cotyledon (RL) in three main categories.

To identify the anthocyanin accumulation genes in the red buckwheat cultivar, the DEGs were evaluated with GO and KEGG pathway analyses. According to GO annotation, the proportions of enriched unigenes were summarized in three main GO categories and distributed into 49 functional terms as follows: 19 terms for biological process, 16 terms for cellular component, and 14 terms for molecular function. These unigenes in the biological process group were mainly involved in the metabolic process, the cellular process, and the single-organism process. The cellular component terms related to cell, cell part, and organelle. Most of the molecular function unigenes were located in the catalytic activity and binding (Figure 4). Meanwhile, an enrichment analysis based on the KEGG database was carried out to further explore the biological functions of the DEGs. The DEGs were significantly enriched in “flavonoid biosynthesis”, “photosynthesis”, and “valine, leucine, and isoleucine biosynthesis” in the WF vs. RF comparison, and “taurine and hypotaurine metabolism”, “photosynthesis”, and “valine, leucine and isoleucine biosynthesis” were significantly enriched in the KEGG pathway analysis in the WL vs. RL comparison (Table S3).

### 2.5. Expression Profile of Genes Responsible for Anthocyanins Production

**Figure 5:**
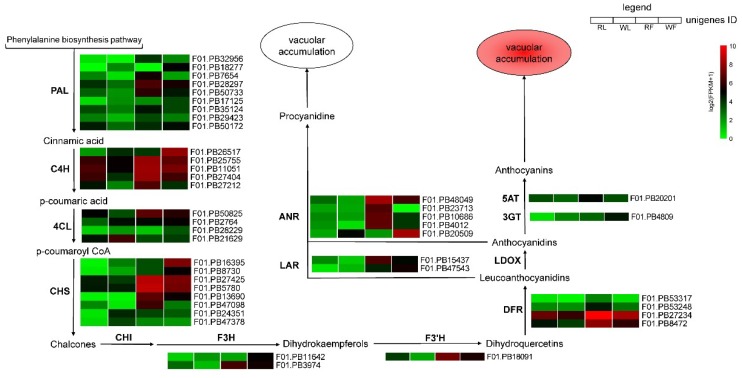
Heat map diagrams of relative expression levels of anthocyanin biosynthesis-related structural genes in the different colors of buckwheat. PAL: Phenylalanine ammonia-lyase, C4H: Cinnamate 4-hydroxylase, 4CL: 4-coumaroyl:CoA ligase, CHS: Chalcone synthase, CHI: Chalcone isomerase, F3H: Flavanone 3-hydroxylase, F3’H: Flavanone 3’-hydroxylase, DFR: Dihydroflavonol 4-reductase, LDOX: Leucoanthocyanidin dioxygenase, LAR: Leucoanthocyanidin reductase, ANR: Anthocyanidin reductase, 3GT: UDP-glucose: Anthocyanidin 3-O-glucosyltransferase, 5AT: Anthocyanin 5-aromatic acyltransferase.

The gene expression levels were normalized to the FPKM (Reads per kilobase of exon model per million mapped reads) to compare the changes in gene expression levels between different colored buckwheat, and the twelve samples were hierarchically clustered based on the log2(FPKM+1), allowing us to observe the overall gene expression pattern (Figure 5). A total of 11 anthocyanin biosynthesis-related structural genes were identified as DEGs, and most of them showed high expression levels in the red buckwheat compared with the white cultivar.

The predicted proteins encoded by upstream genes included nine *PAL* (*phenylalanine ammonia-lyase*) genes that were identified, most of which (except for F01.PB17125 and F01.PB18277) were up-regulated in the RF and RL compared with WF and WL. Five *C4H* (*Cinnamate 4-hydroxylase*) genes were identified, most of which (except for F01.PB26517) were up-regulated in the RF and RL compared with WF and WL. Four *4CL* (*4-coumaroyl:CoA ligase*) genes were identified, among which the expression levels of F01.PB50825 was higher in the red cultivar than the white cultivar, and the rest of them were down-regulated. CHS (chalcone synthase) plays an important role in anthocyanin biosynthesis, and eight *CHS* genes were identified, and half of them were up-regulated in the RF and RL compared with WF and WL. One *F3’H* (*flavanone 3*′*-hydroxylase*) gene and four *DFR* (*dihydroflavonol 4-reductase*) genes identified here displayed similar expression profiles, which were up-regulated in the red cultivar compared with the white cultivar. Additionally, there were no *LDOX* (*leucoanthocyanidin dioxygenase*) genes identified as DEGs, but there were five *ANR* (*anthocyanidin reductase*) genes and two *LAR* (*leucoanthocyanidin reductase*) genes, which can promote PA biosynthesis and contribute to the green skinned variant, were identified, and they (except for F01.PB20509) showed expression patterns similar to those of *F3’H*, except that one *ANR* gene was up-regulated in the white cultivar compared with the red cultivar.

**Figure 6:**
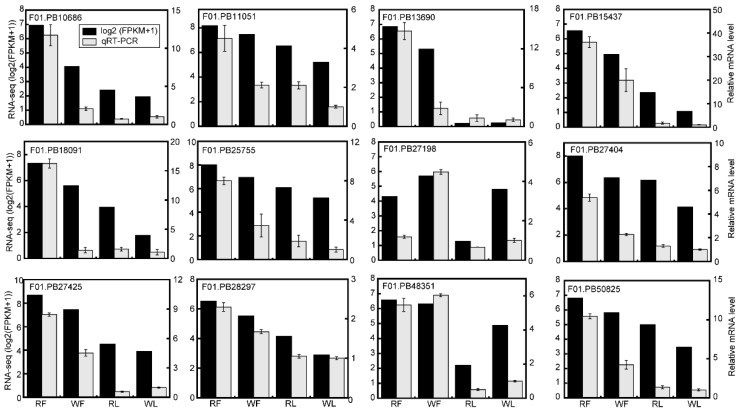
Expression of anthocyanin biosynthesis-related unigenes quantified by RNA-seq (RNA sequencing) and qRT-PCR (Quantitative real-time PCR) analysis. The left y-axis indicates the log2(FPKM+1) values of unigenes from RNA-seq data (black columns). The right y-axis indicates relative gene expression levels analyzed by qRT-PCR (white columns), and the expression of WL was chosen as the control standardizing all samples, values are presented as mean ± SE (*n* = 3).

In order to verify the RNA-Seq data, real-time PCR was carried out to validate the candidate genes involved in the anthocyanin biosynthesis. Thirteen candidate DEGs were selected for RT-qPCR assays. The expression pattern of these genes were consistent with those of the transcriptome analysis (Figure 6).

## 3. Discussion

### 3.1. Components Variations of Anthocyanin and Color Levels Between “HHTQ” and “Beizaosheng”

The types and quantities of anthocyanins differ among different species. Previous studies have reported that cyanidin 3-*O*-gulcoside and cyanidin 3-*O*-rutinoside were identified as the major anthocyanin compound in buckwheat [8,9,31]. In red walnut, the main anthocyanin is cyanidin-3-*O*-galactoside [32], and malvidin-3,5-*O*-diglucoside was detected as the major anthocyanin in grapes [33]. In this study, we detected differences in composition and concentrations of anthocaynins between the cotyledons and flowers of buckwheat. “HHTQ” had shown the highest anthocyanins accumulation in cotyledons and flowers, and the accumulation of cyanidin 3-*O*-rutinoside in flowers and the accumulation of cyanidin 3-*O*-gulcoside in cotyledon are much higher. Meanwhile, anthocyanins remained the lowest in “Beizaosheng”. This result suggests that the pathways of anthocyanin synthesis differ between the cotyledon and flowers of red common buckwheat and confirmed that the phenotypic differences in coloration between red and green common buckwheat are due to the anthocyanin accumulation. These differences were consistent with the different gene expression pattern in the anthocyanin biosynthesis pathway.

### 3.2. Genes Involved in the Anthocyanin Biosynthesis Pathway are Differentially Regulated

The anthocyanin biosynthetic pathway is the branch of the phenylpropanoid and flavonoid pathways, and anthocyanins are synthesized in reactions catalyzed by a series of enzymes. Phenylalanine (Phe), the direct precursors of anthocyanin biosynthesis, is catalyzed by PAL, C4H, and 4CL, converting Phe into p-coumaroyl CoA. CHS, the first committed enzyme in anthocyanin biosynthesis [32], catalyzes the p-coumaroyl CoA to chalcone, while chalcone isomerase (CHI) catalyzes the conversion of chalcone to naringenin. F3H, which belongs to the OGD family [10], catalyzes naringenin to form dihydrokaempferol (DHK). The conversion of DHK to (2*R*,3*R*)-dihydroquercetin and dihydromyricetin is catalyzed by flavonoid 3′-hydroxylase (F3′H) and flavonoid 3′,5′-hydroxylase (F3′5′H), respectively. F3′H and F3′5′H are the key enzymes that determine the structures of anthocyanins and affect color formation [20]. Dihydroflavonol-4-reductase (DFR) catalyzes dihydroflavonols to leucoanthocyanidins, which then are converted to corresponding colored anthocyanidins by LDOX/ANS. Finally, UFGT catalyzes anthocyanidin to anthocyanin, and then transports it to the vacuole. In many plants, the expression levels of certain anthocyanin biosynthetic genes were strongly associated with the anthocyanin accumulation [34,35,36,37]. In this study, we identified 35 DEGs that were annotated as certain anthocyanin biosynthetic genes that were up-regulated in RF and RL. *F3*′*H*, which leads to the production of the red cyanidin-based pigments [38,39,40], and DFR, which is a key enzyme committed to anthocyanin biosynthesis in the flavonoid biosynthetic pathway [7,41], displayed significantly increased expression levels in RF (Figure 5). Notably, these results suggested that these unigenes are responsible for color formation in the cotyledons and flowers of “HHTQ”.

However, none of the DEGs were annotated as *CHI* and *F3′5′H*, except for the DEG, F01.PB27425, which was annotated as *CHS*, and showed significantly higher expression levels in the RF than WF. Meanwhile, another DEG, F01.PB16395, was annotated as *CHS* and showed higher transcriptional levels in “Beizaosheng” than in “HHTQ” (Figure 5). Li et al. [32] has also detected three unigenes (annotated as *CHS*, *F3H*, and *F3’5’H*, respectively), demonstrating significantly higher expression levels in green peel than in the red peel of walnut, of which the green tissues are expected to contain more colorless flavonoids.

A critical step in anthocyanin synthesis is mediated by dihydroflavonol-4-reductase (DFR), and overexpression of *DRF* can increases the accumulation of anthocyanins and produce red flowers [42]. In the present study, four *DFR* genes were identified and the F01.PB27234 was mostly expressed in the RF (FPKM > 1000), which indicates that it may be crucial for anthocyanin accumulation in the red flowers of buckwheat. Anthocyanidin 3-*O*-glucosyltransferase (3GT) catalyzes the transfer of the glucosyl moiety form UDP-glucose to the 3-hydroxyl group of the corresponding acceptor molecule. This glycosylation step play an important role in anthocyanin stability and water solubility [43,44]. In our transcriptome analysis, one unigene (F01.PB20201), annotated as *3GT*, was found to be lower expressed in the flowers of the “HHTQ” compared with the “Beizaosheng” cultivar. F01.PB20201, annotated as anthocyanin 5-aromatic acyltransferase (5AT), which acrylates the glucose bound at the position of anthocyanidin 3,5-diglucoside [45], was up-regulated in RF. These genes may be involved in the anthocyanin biosynthesis and may cause the formation of red flower buckwheat. These results also point out the direction for further studies on the coloration of vegetables and fruit.

### 3.3. Components DEGs Related to the Anthocyanin Degradation

Leucoanthocyanidin reductase (LAR) can convert leucoanthocyanidin to 2,3-*trans*-flavanols, such as (+)-catechin, and its activity correlated with proanthocyanidins accumulation [46,47]. Previous studies indicated that ectopic expression of the apple *MdLAR1* gene in tobacco inhibits expression of the late genes in anthocyanin biosynthetic pathways, resulting in a decrease in anthocyanin accumulation in flowers [48]. In our transcriptome data, we also noticed that one *LAR* gene (F01.PB47543) was up-regulated in the “Beizaosheng” compared with “HHTQ”. The *ANR* gene encodes an anthocyanin reductase that can reduce anthocyanidins to flavan-3-ols required for the formation of proanthocyanidins on the flavonoids pathway [49,50]. In the present study, four of the *ANR* genes were up-regulated in the RF, however, one *ANR* gene (F01.PB20509) was down-regulated in the flowers and cotyledons of “HHTQ”. The previous study showed that experimental down-regulation of *ANR* by *RNAi* can induce premature and ectopic anthocyanin formation in *Fragaria* × *ananassa* [51]. Whether these genes are involved in the anthocyanin degradation and exert the same effect as the homologous genes still needs further analysis.

### 3.4. DEGs Involved in the Regulation of Anthocyanin Accumulation

Additionally, anthocyanin accumulation is also regulated by a suite of transcription factors (TFs), including MYB, bHLH, and WD40 families [52,53,54]. The MYB and bHLH proteins combine with the WD40 protein to form WD-repeat/Myb/bHLH transcriptional complexes that activate anthocyanin biosynthetic genes [25,55,56], and the R2R3-MYB can predominantly regulate the accumulation of anthocyanin [18]. In this study a *MYB1* homolog (F01.PB20382) and a *TT2* homolog (F01.PB13850) were identified and showed a high transcription level in the flower of “HHTQ”. Additionally, three *bHLH143* (F01.PB46560, F01.PB12993, and F01.PB52152) were also up-regulation in the RF compared with WF (Table S4). These findings suggest that the MYB and bHLH family may correlate with the regulation of anthocyanin accumulation in the buckwheat. On the other hand, phytohormones, such as abscisic acid (ABA) and jasmonates (JAs), can also stimulate anthocyanin accumulation by regulation anthocyanin biosynthetic genes [57,58,59,60,61]. In sweet cherry (*Prunus avium* L.), ABA treatment strongly induced anthocyanin accumulation and the colorless phenotype was also observed in the fruits when silencing *PacNCED1* (*9-cis-epoxycarotenoid dioxygenase 1*), the cognate gene encoding a key enzyme in the ABA biosynthesis pathway [59]. In addition, previous studies have shown that JA can strongly induce the LBGs in *Arabidopsis* [57], and the jasmonate-ZIM-domain (JAZ) proteins, acting as negative regulators of jasmonate-responsive genes, degrade in response to the JA signal, which releases bHLH and MYB TFs to repress JA-regulated anthocyanin accumulation [58,62,63]. In this study, we also detected several *NCED1* homologs and JAZ protein homologs (Table S5), and most of the *NCED1* homologs showed no significant change between red and white cultivar, however, four JAZ proteins were identified and showed down-regulated in the red buckwheat. These results suggest that the *NCED1* genes may not associate with anthocyanin accumulation in the buckwheat, but the JAZ proteins might act as negative regulators and be involved in the anthocyanin biosynthesis pathway. These homologous genes still need further analysis.

## 4. Materials and Methods 

### 4.1. Plant Materials

Two cultivars, the red common buckwheat ‘HHTQ’ (red leaves and red flowers), and the green common buckwheat ‘Beizaosheng’ (green leaves and white flowers), were used in this study. They were grown in fields at Yangtze University, Jinzhou, China. When the flowers appeared, flower tissues from both cultivars were collected. Cotyledons were collected from 7-day-old aseptic seedlings, which were grown in sugar-free MS medium. All samples were quickly frozen in liquid nitrogen and stored at –80°C until used. To investigate the accumulation pattern of anthocyanin, different growth stages of flowers and leaves were harvested and mixed to measured anthocyanin content. Leaves were classified according to leaf width: “cotyledons”: 4.0–5.0 cm, “Stage 1”: 4.0–5.0 cm, “Stage 2”: 5.0–6.0 cm, “Stage 3”: 6.0–7.0 cm, “Stage 4”: 7.0–9.0 cm, and fresh weights were used to classify the flowers as described by Suzuki et al. [8]: “Stage 1”: 0.0–0.4 mg, “Stage 2”: 0.4–0.8 mg, “Stage 3”: 0.8–1.6 mg, “Stage 4”: 1.6–2.6 mg, “Stage 5” (fully developed petal): 2.6–3.6 mg.

### 4.2. RNA Preparation

The cotyledons and flowers tissues were collected from red cultivar ‘HHTQ’ and white cultivar ‘Beizaosheng’. The total RNA was extracted from the samples using EASYspin Plant RNA Kit (Aidlab, Wuhan, China) according to the manufacturer’s instructions, and were then treated with DNase I to remove genomic DNA [64]. RNA quality was checked and quantified using a Nanodrop 2000r (Nanodrop Technologies, Wilmington, DE, USA) and Agilent bioanalyzer 2100 (Agilent Technologies, Santa Clara, CA, USA), respectively.

### 4.3. PacBio Iso-Seq Library Preparation and Sequencing

Full-length transcriptome sequencing was carried out at Biomarker Technologies Co. Ltd. (Beijing, China), following manufacturer protocols. Briefly, the high-quality, full-length cDNAs was synthesized from total RNA by using a SMARTer™ PCR cDNA Synthesis Kit (Takara, Dalian, China), and after a round of PCR amplification, the BluePippin™ Size Selection System (Sage Science, Beverly, MA, USA) was used to construct cDNA libraries of different sizes: 1–2 K, 2–3 K, and 3–6 K. Three Iso-Seq libraries were sequenced on five cells with PacBio RS II, and Illumine sequencing data were used to polish the PacBio assembly. The raw sequencing data were submitted to the National Center for Biotechnology Information (NCBI) Sequence Read Archive with a Bioproject ID: PRJNA517031.

### 4.4. Illumina Sequencing, Data Assembly, and Gene Annotation

Illumina sequencing reads analysis was performed at Biomarker Technologies Co. Ltd. (Beijing, China), following manufacturer protocols. Each sample has three replicates. Briefly, mRNA was separated from total RNA using oligo (dT) magnetic beads in accordance with the Illumina manufacturer’s instructions and fragmented into small pieces using a fragmentation buffer. Then, the first- and second- stand cDNA were synthesized using these fragments as reverse transcription templates. Finally, paired-end adapters were ligated to the ends of the cDNA fragments. The appropriate DNA fragments were used as amplification templates for polymerase chain reaction amplification. The cDNA libraries were sequenced on Illumina HiSeq 2500 platform. The raw reads were submitted to the National Center for Biotechnology Information (NCBI) Sequence Read Archive with a Bioproject ID: PRJNA517026.

Quality parameters, including GC-content, sequence duplication level, and Q30 were used to filter the reads from the Illumina RNA-seq data. Then, the high-quality clean data were obtained from the raw reads after removing the low-quality data, which included adapter sequences and low-quality reads. Cleaned and qualified reads were assembled in Trinity software [65], and the resulting sequences were called Unigenes. BLASTX alignment was performed between these unigene sequences and public protein databases: the NCBI non-redundant (Nr), Swiss-Prot, clusters of orthologous groups of proteins (COG), euKaryotic ortholog groups (KOG), protein family (Pfam), gene ontology (GO), Kyoto encyclopedia of genes and genomes (KEGG) database, and nucleotide database (NT) [66]. Cut-off value was set as e-value < 1 × 10^−5^.

### 4.5. Differentially Expressed Gene Identification

For differential expression tests (DET), we compared two conditions: WF vs. RF, and WL vs. RL. For each comparison, genes with low read counts per million (cpm < 1) that did not represent sufficient statistical significance were excluded from the DET analysis. The libraries were digitally normalized after filtering using the DESeq. Genes with |Fold Change| ≥ 2 and FDR (false discovery rate) < 0.01 were considered as highly differentially expressed genes (DEGs). The gene expression unit was calculated using the FPKM method (fragments per kilobase of transcript per million mapped reads).

### 4.6. GO Functional Enrichment and Pathway Analysis of DEGs

The DEGs were mapped to GO terms in the GO database (http://www.geneontology.org/) to calculate a gene number for every term. The hypergeometric test was used to find significantly enriched GO terms based on ‘GO::TermFinder’ (http://smd.stanford.edu/help/GOTermFinder/GO_TermFinder_help.shtml/). GO terms conforming top-value through Bonferroni Correction ≤ 0.05 were defined as significantly enriched GO terms. KEGG (http://www.genome.jp/kegg/) was used to perform pathway enrichment analysis of DEGs. MapMan software (version 3.6.0) was used to display expression profiles at the pathway level.

### 4.7. Quantitative Real-Time PCR (qRT-PCR) Analysis

Total RNA from flowers and cotyledons of both cultivars were extracted and subjected to DNase I digestion (Takara, Dalian, China) to remove DNA. The first-strand cDNA for qPCR analysis was synthesized from 500 ng of total RNA using oligo dT (Takara, Dalian, China) according to the manufacturer’s protocols. The primers were designed using Primer Premier 5.0 and beta-Actin was used as a reference gene (Table S6). The qRT-PCR was performed on a CFX 96 real-time PCR system (BioRad, Hercules, CA, USA) using SYBR (TaKaRa), according to manufacturer’s protocols. Each treatment included three replications [67].

### 4.8. Extraction and HPLC Analysis of Anthocyanins

Anthocyanins were extracted and determined according to published methods with minor modifications [68,69]. Different stages of flower and leaf tissues were collocated and mixed to measure the anthocyanin content. Briefly, 0.1 g of dry samples were extracted with 5 mL of acidic methanol containing 1% HCl (*v*/*v*) in the dark at 4 °C overnight, with occasional shaking. Plant materials were sedimented by centrifugation at 4450× *g* for 15 min, and 500 μL of the supernatant was mixed with 500 μL of MilliQ H_2_O and 300 μL of chloroform in a 1.5-mL microcentrifuge tube to remove chlorophyll in the extraction. After centrifugation at 12,000× *g* for 5 min, the supernatants containing anthocyanins were filtered through a Millipore filter (0.22 mm diameter). The HP1260-VWD system (Agilent Technologies) was used to analyze anthocyanins in the extract at 520 nm. A GL Sciences Inertsil ODS-3 C18 column (250 mm × 4.6 mm, 5 μm) was used for separation at 25 °C and eluted using a mobile phase consisting of solvent A (methanol) and solvent B (5% formic acid in water) at a flow rate of 1 mL/min. The elution program was as follows: 0–2 min, 10% A; 2–6 min, 10–20% A; 6–10 min, 20–30% A; 10–15 min, 30–35% A; 15–20 min, 35–50% A; 20–24 min, 50–90% A; 24–29 min, 90–10% A; 29–30 min, 10% A. The anthocyanin standards, cyanidin 3-*O*-glucoside and cyanidin 3-*O*-rutinoside were used as controls and to generate standard curves for quantitative analysis of the buckwheat samples.

### 4.9. Statistical Treatment

All experiments were carried out in triplicate, and data were expressed as mean ± SD (standard deviation). Statistical significance was determined by one-way ANOVA, and statistical significance was accepted at a *p*-value threshold of <0.05.

## 5. Conclusions

A high level of anthocyanin accumulation was observed in the cotyledons and flowers of “HHTQ”, and candidate genes involved in the anthocyanin biosynthetic pathway were identified using transcriptome analysis. A total of 3727 and 5323 DEGs were identified in flowers and cotyledons, respectively. The putative structural genes and regulatory genes involved in the regulation of anthocyanin biosynthesis by the different expression patterns, and anthocyanin degradation enzymes, such as ANR and LAR, might play a critical role in degrading anthocyanin. These data will point out the direction for further study on coloration and contribute to a more thorough characterization of anthocyanin biosynthesis at the molecular level in buckwheat. Finally, the red common buckwheat material could be used for hybrid breeding and the genes that are connecting with color formation could be used to improve the anthocyanin content in buckwheat.

## Figures and Tables

**Table 1:** Anthocyanin levels (mg/g dry weight) in different tissues of the two buckwheat cultivars.

Peak	Compound	Samples			
		RF	RL	WF	WL
1	cyanidin 3-*O*-gulcoside	0.52 ± 0.01	0.97 ± 0.02	0.07 ± 0.01	0.26 ± 0.01
2	cyanidin 3-*O*-rutinoside	2.83 ± 0.01	0.36 ± 0.01	0.38 ± 0.02	Nd ^1^
Total		3.38 ± 0.01	1.33 ± 0.02	0.45 ± 0.03	0.26 ± 0.01

^1^ Nd means not detected.

## Supplementary Materials

Supplementary materials can be found at https://www.mdpi.com/1422-0067/20/6/1493/s1.

## Abbreviations

HPLC	High performance liquid chromatography
DET	Differentially expression test
DEGs	Differently expressed genes
EBGs	Early-stage biosynthetic genes
LBGs	Late-stage biosynthetic genes
GO	Gene ontology
KEGG	Kyoto encyclopedia of genes and genomes
DHK	Dihydrokaempferol
